# Anilinium-3-carboxyl­ate 3-carb­oxy­anilinium nitrate

**DOI:** 10.1107/S1600536812049392

**Published:** 2012-12-08

**Authors:** Saeed Ahmad, Sajjad Hussain, Shahzad Sharif, Islam Ullah Khan, Muhammad Nawaz Tahir

**Affiliations:** a Department of Chemistry, University of Engineering and Technology, Lahore 54890, Pakistan; b Department of Chemistry, Government College University, Lahore, Pakistan; c Department of Physics, University of Sargodha, Sagrodha, Pakistan

## Abstract

The title compound, C_7_H_8_NO_2_^+^·NO_3_^−^·C_7_H_7_NO_2_, exists in the form of a protonated dimer of two anilinium-3-carboxyl­ate mol­ecules related by an inversion center, and a nitrate anion located on a twofold rotation axis. The bridging H atom occupies, with equal probability, the two sites associated with the carboxyl atoms. In addition to the strong O—H⋯O hydrogen bond, in the crystal, the various units are linked *via* N—H⋯O and C—H⋯O hydrogen bonds forming a three-dimensional structure.

## Related literature

For applications of amino­benzoic acids, see: Congiu *et al.* (2005 ▶); Swislocka *et al.* (2005 ▶). For related structures and details of their hydrogen-bonding motifs, see: Arora *et al.* (1973 ▶); Bahadur *et al.* (2007 ▶); Hansen *et al.* (2007 ▶); Lai & Marsh (1967 ▶); Lu *et al.* (2001 ▶); Smith *et al.* (1995 ▶); Zaidi *et al.* (2008 ▶).
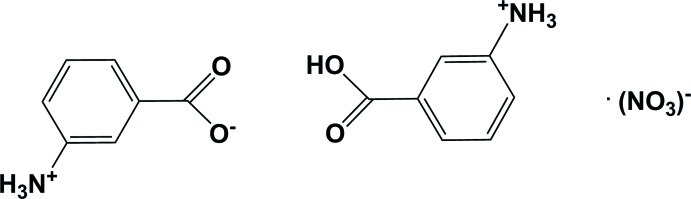


## Experimental

### 

#### Crystal data

C_7_H_8_NO_2_^+^·NO_3_^−^·C_7_H_7_NO_2_
*M* *_r_* = 337.29
Monoclinic, 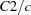

*a* = 16.0451 (3) Å
*b* = 4.7575 (1) Å
*c* = 19.7143 (4) Å
β = 107.660 (1)°
*V* = 1433.96 (5) Å^3^
*Z* = 4
Mo *K*α radiation
μ = 0.13 mm^−1^
*T* = 296 K
0.23 × 0.16 × 0.07 mm

#### Data collection

Bruker Kappa APEXII CCD area-detector diffractometer
Absorption correction: multi-scan (*SADABS*; Sheldrick, 1996 ▶) *T*_min_ = 0.525, *T*_max_ = 0.806
6955 measured reflections
1777 independent reflections
1604 reflections with *I* > 2σ(*I*)
*R*_int_ = 0.017

#### Refinement

*R*[*F* ^2^ > 2σ(*F* ^2^)] = 0.037
*wR*(*F* ^2^) = 0.101
*S* = 1.07
1777 reflections
127 parameters
H atoms treated by a mixture of independent and constrained refinement
Δρ_max_ = 0.29 e Å^−3^
Δρ_min_ = −0.20 e Å^−3^

### 

Data collection: *APEX2* (Bruker, 2007 ▶); cell refinement: *SAINT* (Bruker, 2007 ▶); data reduction: *SAINT*; program(s) used to solve structure: *SHELXS97* (Sheldrick, 2008 ▶); program(s) used to refine structure: *SHELXL97* (Sheldrick, 2008 ▶); molecular graphics: *PLATON* (Spek, 2009 ▶); software used to prepare material for publication: *SHELXL97*, *PLATON* and *publCIF* (Westrip, 2010 ▶).

## Supplementary Material

Crystal structure: contains datablock(s) I, global. DOI: 10.1107/S1600536812049392/kj2215sup1.cif

Structure factors: contains datablock(s) I. DOI: 10.1107/S1600536812049392/kj2215Isup2.hkl

Supplementary material file. DOI: 10.1107/S1600536812049392/kj2215Isup3.cml

Additional supplementary materials:  crystallographic information; 3D view; checkCIF report

## Figures and Tables

**Table 1:** Hydrogen-bond geometry (Å, °)

*D*—H⋯*A*	*D*—H	H⋯*A*	*D*⋯*A*	*D*—H⋯*A*
O1—H1*O*⋯O1^i^	0.88 (4)	1.61 (4)	2.4868 (13)	171 (4)
O1—H1*O*⋯O2^i^	0.88 (4)	2.55 (3)	3.0686 (14)	118 (3)
N1—H1*N*⋯O2^ii^	0.913 (18)	2.027 (19)	2.9157 (13)	164.2 (17)
N1—H2*N*⋯O2^iii^	0.944 (18)	1.918 (18)	2.8609 (13)	177.0 (16)
N1—H3*N*⋯O3^iv^	0.939 (19)	2.498 (15)	2.9220 (14)	107.6 (11)
N1—H3*N*⋯O3	0.939 (19)	2.526 (15)	2.9582 (14)	108.3 (11)
N1—H3*N*⋯O4	0.939 (19)	1.920 (19)	2.8345 (11)	163.9 (13)
C3—H3⋯O2^ii^	0.93	2.41	3.1189 (15)	132
C5—H5⋯O3^iv^	0.93	2.58	3.3058 (18)	135

Symmetry codes: (i) 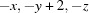; (ii) 
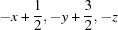
; (iii) 
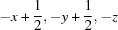
; (iv) 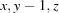.

**Fig. 1.**
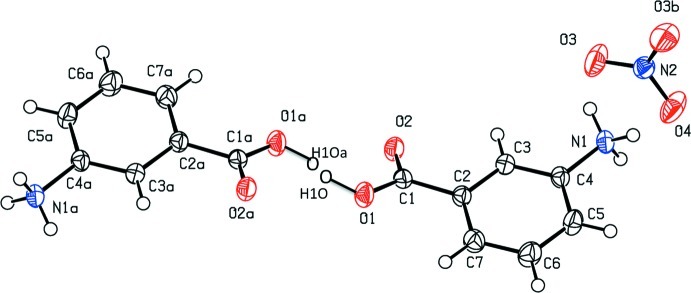
A view of the molecular structure of the title compound, with the atom numbering. Displacement ellipsoids are drawn at the 50% probability level. Atom H1O has an occupancy of 0.5 [symmetry codes: (a) = -x, -y + 2, -z; (b) -x + 1, y, -z + 1/2].

**Fig. 2.**
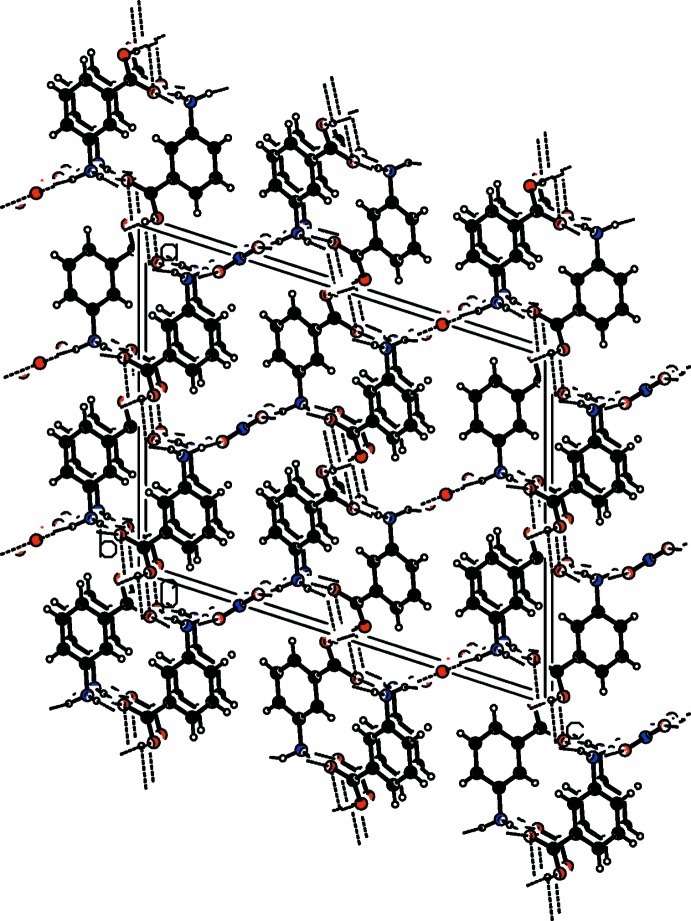
A view along the b axis of the crystal packing of the title compound. Hydrogen bonds are shown as dashed lines - see Table 1 for details [C-bound H atoms have been omitted for clarity].

## supplementary crystallographic information

### Comment

Amino derivatives of benzoic acid are of considerable importance because of their use as anti-inflammatory and anticancer agents (Congiu *et al.*, 2005). Benzoic acid, its derivatives and their complexes are also used as food preservatives and as antiseptic agents applied in various industrial branches: pharmaceutics, textile and cosmetics (Swislocka *et al.*, 2005). In view of this interest, the crystal structures of various amino derivatives of benzoic acid (Hansen *et al.*, 2007; Lai *et al.*, 1967; Lu *et al.*, 2001; Smith *et al.*, 1995), and their ammonium salts (Arora *et al.*, 1973; Bahadur *et al.*, 2007; Zaidi *et al.*, 2008), have been reported in the literature. The crystal structures of these compounds are characterized by strong hydrogen bonding.

The ammonium salts of 2-aminobenzoic acid are monomers (Bahadur *et al.*, 2007; Zaidi *et al.*, 2008), whereas the chloride salt of the anilinium-3-carboxylate ion (Arora *et al.*, 1973) exists in the form of hydrogen-bonded dimers formed through the carboxylic acid groups of inversion related molecules. In the present study, we attempted to prepare a cerium(III) complex of 3-aminobenzoic acid but the resulting product was a simple nitrate salt of the acid. Herein, we present the crystal structure of this salt.

In the title compound two anilinium-3-carboxylate molecules related by an inversion center are bound to a proton to form a protonated dimer through strong O—H···O hydrogen bonds (Fig. 1 and Table 1). A nitrate anion located on a 2-fold rotation axis is present as counter ion. The bridging H atom (H1O) occupies, with equal probability, the two sites associated with the carboxyl atoms, O1 and O1a [symmetry code: (*a*) = -*x*, -*y* + 2, -*z*]. The ammonium groups are involved in strong hydrogen bonds to the carbonyl as well as to the nitrate O atoms (Table 1).

In the crystal, (Fig. 2 and Table 1) molecules are linked *via* N—H..O and C—H..O hydrogen bonds forming a three-dimensional structure.

### Experimental

The title compound was prepared by adding one equivalent of 3-aminobenzoic acid (0.07 g) in 15 ml methanol to a solution of cerium nitrate (0.22 g, 0.5 mmol) in 15 ml me thanol. The brown solution was stirred for one hour, after which it was filtered and the filtrate was kept for crystallization at room temperature. The solution was covered with aluminium foil. After 3 days large orange-brown crystals were obtained (*M*.p. = 492 (1) K). A plate-shaped fragment cut from a large crystal was used for data collection.

### Refinement

The OH H atom was located in a difference Fourier map and refined freely with a fixed occupancy of 0.5. The NH_3_ atoms were located from a difference Fourier map and refined freely. The C—H atoms were placed in calculated positions and treated as riding atoms: C—H = 0.93 Å with *U*_iso_(H) = 1.2*U*_eq_(C).

### Crystal data

**Table:**

C_7_H_8_NO_2_^+^·NO_3_^−^·C_7_H_7_NO_2_	*F*(000) = 704
*M**_r_* = 337.29	*D*_x_ = 1.562 Mg m^−^^3^
Monoclinic, *C*2/*c*	Mo *K*α radiation, λ = 0.71073 Å
Hall symbol: -C 2yc	Cell parameters from 1777 reflections
*a* = 16.0451 (3) Å	θ = 2.9–28.3°
*b* = 4.7575 (1) Å	µ = 0.13 mm^−^^1^
*c* = 19.7143 (4) Å	*T* = 296 K
β = 107.660 (1)°	Plate, pale orange
*V* = 1433.96 (5) Å^3^	0.23 × 0.16 × 0.07 mm
*Z* = 4	

### Data collection

**Table:**

Bruker Kappa APEXII CCD area-detector diffractometer	1777 independent reflections
Radiation source: fine-focus sealed tube	1604 reflections with *I* > 2σ(*I*)
Graphite monochromator	*R*_int_ = 0.017
ω scans	θ_max_ = 28.3°, θ_min_ = 3.9°
Absorption correction: multi-scan (*SADABS*; Sheldrick, 1996)	*h* = −20→21
*T*_min_ = 0.525, *T*_max_ = 0.806	*k* = −6→6
6955 measured reflections	*l* = −26→24

### Refinement

**Table:**

Refinement on *F*^2^	Secondary atom site location: difference Fourier map
Least-squares matrix: full	Hydrogen site location: inferred from neighbouring sites
*R*[*F*^2^ > 2σ(*F*^2^)] = 0.037	H atoms treated by a mixture of independent and constrained refinement
*wR*(*F*^2^) = 0.101	*w* = 1/[σ^2^(*F*_o_^2^) + (0.0497*P*)^2^ + 1.028*P*] where *P* = (*F*_o_^2^ + 2*F*_c_^2^)/3
*S* = 1.07	(Δ/σ)_max_ < 0.001
1777 reflections	Δρ_max_ = 0.29 e Å^−^^3^
127 parameters	Δρ_min_ = −0.20 e Å^−^^3^
0 restraints	Extinction correction: *SHELXL97* (Sheldrick, 2008), Fc^*^=kFc[1+0.001xFc^2^λ^3^/sin(2θ)]^-1/4^
Primary atom site location: structure-invariant direct methods	Extinction coefficient: 0.0050 (11)

### Special details

**Table:**

Geometry. Bond distances, angles *etc*. have been calculated using the rounded fractional coordinates. All su's are estimated from the variances of the (full) variance-covariance matrix. The cell e.s.d.'s are taken into account in the estimation of distances, angles and torsion angles
Refinement. Refinement of *F*^2^ against ALL reflections. The weighted *R*-factor *wR* and goodness of fit *S* are based on *F*^2^, conventional *R*-factors *R* are based on *F*, with *F* set to zero for negative *F*^2^. The threshold expression of *F*^2^ > σ(*F*^2^) is used only for calculating *R*-factors(gt) *etc*. and is not relevant to the choice of reflections for refinement. *R*-factors based on *F*^2^ are statistically about twice as large as those based on *F*, and *R*- factors based on ALL data will be even larger.

### Fractional atomic coordinates and isotropic or equivalent isotropic displacement parameters (Å^2^)

**Table:**

	*x*	*y*	*z*	*U*_iso_*/*U*_eq_	Occ. (<1)
O1	0.03166 (6)	0.8054 (2)	0.04202 (5)	0.0316 (3)	
O2	0.11051 (6)	0.75160 (18)	−0.03226 (4)	0.0292 (3)	
N1	0.38230 (6)	0.2414 (2)	0.11339 (6)	0.0256 (3)	
C1	0.09454 (7)	0.6963 (2)	0.02418 (6)	0.0232 (3)	
C2	0.15270 (7)	0.4950 (2)	0.07568 (6)	0.0234 (3)	
C3	0.23625 (7)	0.4507 (2)	0.07021 (6)	0.0241 (3)	
C4	0.29346 (7)	0.2769 (2)	0.11825 (6)	0.0232 (3)	
C5	0.26851 (8)	0.1375 (3)	0.17048 (6)	0.0316 (3)	
C6	0.18493 (9)	0.1785 (3)	0.17490 (7)	0.0355 (4)	
C7	0.12742 (8)	0.3602 (3)	0.12863 (7)	0.0308 (3)	
O3	0.44638 (8)	0.7461 (2)	0.20141 (6)	0.0512 (4)	
O4	0.50000	0.3567 (3)	0.25000	0.0540 (5)	
N2	0.50000	0.6216 (3)	0.25000	0.0283 (4)	
H1N	0.3935 (11)	0.384 (4)	0.0864 (9)	0.045 (4)*	
H1O	0.014 (2)	0.953 (8)	0.0144 (17)	0.032 (8)*	0.500
H2N	0.3850 (11)	0.075 (4)	0.0881 (9)	0.044 (4)*	
H3	0.25330	0.53810	0.03430	0.0290*	
H3N	0.4235 (12)	0.243 (3)	0.1589 (10)	0.043 (4)*	
H5	0.30730	0.01800	0.20220	0.0380*	
H6	0.16720	0.08300	0.20930	0.0430*	
H7	0.07210	0.39160	0.13310	0.0370*	

### Atomic displacement parameters (Å^2^)

**Table:**

	*U*^11^	*U*^22^	*U*^33^	*U*^12^	*U*^13^	*U*^23^
O1	0.0287 (4)	0.0321 (5)	0.0368 (5)	0.0116 (4)	0.0142 (4)	0.0065 (4)
O2	0.0284 (4)	0.0300 (5)	0.0308 (4)	0.0075 (3)	0.0113 (3)	0.0065 (3)
N1	0.0214 (5)	0.0265 (5)	0.0268 (5)	0.0049 (4)	0.0040 (4)	0.0010 (4)
C1	0.0201 (5)	0.0209 (5)	0.0281 (5)	0.0006 (4)	0.0064 (4)	−0.0005 (4)
C2	0.0222 (5)	0.0215 (5)	0.0257 (5)	0.0022 (4)	0.0059 (4)	−0.0005 (4)
C3	0.0232 (5)	0.0232 (5)	0.0254 (5)	0.0021 (4)	0.0068 (4)	0.0023 (4)
C4	0.0214 (5)	0.0223 (5)	0.0247 (5)	0.0023 (4)	0.0050 (4)	−0.0022 (4)
C5	0.0334 (6)	0.0322 (6)	0.0281 (6)	0.0089 (5)	0.0078 (5)	0.0077 (5)
C6	0.0390 (7)	0.0392 (7)	0.0321 (6)	0.0062 (6)	0.0164 (5)	0.0117 (5)
C7	0.0275 (6)	0.0335 (6)	0.0340 (6)	0.0044 (5)	0.0132 (5)	0.0038 (5)
O3	0.0579 (7)	0.0333 (6)	0.0462 (6)	0.0102 (5)	−0.0086 (5)	0.0069 (5)
O4	0.0663 (10)	0.0240 (7)	0.0470 (9)	0.0000	−0.0196 (7)	0.0000
N2	0.0291 (7)	0.0251 (7)	0.0286 (7)	0.0000	0.0056 (6)	0.0000

### Geometric parameters (Å, º)

**Table:**

O1—C1	1.2753 (15)	C2—C3	1.3932 (17)
O2—C1	1.2434 (14)	C2—C7	1.3865 (17)
O1—H1O	0.88 (4)	C3—C4	1.3769 (15)
O3—N2	1.2283 (13)	C4—C5	1.3822 (17)
O4—N2	1.260 (2)	C5—C6	1.384 (2)
N1—C4	1.4671 (16)	C6—C7	1.386 (2)
N1—H2N	0.944 (18)	C3—H3	0.9300
N1—H3N	0.939 (19)	C5—H5	0.9300
N1—H1N	0.913 (18)	C6—H6	0.9300
C1—C2	1.4980 (15)	C7—H7	0.9300
			
C1—O1—H1O	107 (2)	C2—C3—C4	119.60 (10)
C4—N1—H3N	110.7 (12)	C3—C4—C5	121.15 (11)
H1N—N1—H2N	105.5 (16)	N1—C4—C3	118.84 (10)
C4—N1—H2N	109.4 (11)	N1—C4—C5	120.01 (10)
H2N—N1—H3N	112.3 (14)	C4—C5—C6	118.98 (12)
H1N—N1—H3N	110.2 (15)	C5—C6—C7	120.73 (13)
C4—N1—H1N	108.6 (12)	C2—C7—C6	119.71 (12)
O3^i^—N2—O4	118.83 (8)	C2—C3—H3	120.00
O3—N2—O4	118.83 (8)	C4—C3—H3	120.00
O3—N2—O3^i^	122.34 (13)	C6—C5—H5	120.00
O2—C1—C2	119.12 (10)	C4—C5—H5	121.00
O1—C1—C2	117.10 (10)	C5—C6—H6	120.00
O1—C1—O2	123.76 (10)	C7—C6—H6	120.00
C3—C2—C7	119.77 (10)	C2—C7—H7	120.00
C1—C2—C3	117.42 (10)	C6—C7—H7	120.00
C1—C2—C7	122.79 (11)		
			
O1—C1—C2—C3	−158.16 (10)	C3—C2—C7—C6	−1.16 (18)
O1—C1—C2—C7	20.10 (16)	C2—C3—C4—N1	−177.64 (9)
O2—C1—C2—C3	20.63 (15)	C2—C3—C4—C5	2.25 (16)
O2—C1—C2—C7	−161.12 (11)	N1—C4—C5—C6	178.75 (11)
C1—C2—C3—C4	177.24 (9)	C3—C4—C5—C6	−1.13 (18)
C7—C2—C3—C4	−1.08 (16)	C4—C5—C6—C7	−1.2 (2)
C1—C2—C7—C6	−179.38 (11)	C5—C6—C7—C2	2.3 (2)

Symmetry code: (i) −*x*+1, *y*, −*z*+1/2.

### Hydrogen-bond geometry (Å, º)

**Table:**

*D*—H···*A*	*D*—H	H···*A*	*D*···*A*	*D*—H···*A*
O1—H1*O*···O1^ii^	0.88 (4)	1.61 (4)	2.4868 (13)	171 (4)
O1—H1*O*···O2^ii^	0.88 (4)	2.55 (3)	3.0686 (14)	118 (3)
N1—H1*N*···O2^iii^	0.913 (18)	2.027 (19)	2.9157 (13)	164.2 (17)
N1—H2*N*···O2^iv^	0.944 (18)	1.918 (18)	2.8609 (13)	177.0 (16)
N1—H3*N*···O3^v^	0.939 (19)	2.498 (15)	2.9220 (14)	107.6 (11)
N1—H3*N*···O3	0.939 (19)	2.526 (15)	2.9582 (14)	108.3 (11)
N1—H3*N*···O4	0.939 (19)	1.920 (19)	2.8345 (11)	163.9 (13)
C3—H3···O2^iii^	0.93	2.41	3.1189 (15)	132
C5—H5···O3^v^	0.93	2.58	3.3058 (18)	135

Symmetry codes: (ii) −*x*, −*y*+2, −*z*; (iii) −*x*+1/2, −*y*+3/2, −*z*; (iv) −*x*+1/2, −*y*+1/2, −*z*; (v) *x*, *y*−1, *z*.
